# Dental lessons learned from large-scale military dental records in the Israel defense forces: military dentistry as a window into national oral health trends

**DOI:** 10.1186/s13584-025-00712-5

**Published:** 2025-08-12

**Authors:** Yehuda Zadik, Guy Tobias

**Affiliations:** 1 Saligman Clinics, and Department of Oral Medicine, Faculty of Dental Medicine, The Hebrew University of Jerusalem, and Hadassah Medical Center, Jerusalem, Israel; 2 Department of Community Dentistry, Faculty of Dental Medicine, The Hebrew University of Jerusalem, and Hadassah Medical Center, Jerusalem, Israel

**Keywords:** Aggressive periodontitis, Dental caries, Military medicine, Oral health, Prevention, Public health

## Abstract

Mandatory military service creates a unique opportunity to examine health patterns in a near-comprehensive national cohort of young adults. In this context, large-scale, routinely collected military health data, particularly from dental records, can serve as a powerful lens through which to explore broader public health phenomena. Recent population-based studies conducted within the Israel Defense Forces have demonstrated how clinical records originally intended to ensure operational readiness can also illuminate associations between health outcomes and social determinants. These findings highlight the untapped potential of military health systems to contribute not only to force fitness, but to national health surveillance and epidemiologic insight. Particularly in the realm of oral health, where disease burden remains relatively high and disparities are both widespread and insufficiently explored, military data provide a scalable, real-world foundation for risk assessment, preventive strategies, and informed policy development. The integration of clinical, cognitive, and demographic data from this setting underscores the relevance of military research far beyond its operational context, informing strategies that bridge public health, clinical practice, and social equity.

## Commentary

Dental caries remains among the most prevalent chronic diseases worldwide, including in Israel [1, 2]. Its consequences span a broad spectrum, from impaired oral function, aesthetics, and quality of life to severe life-threatening infections, rendering it a substantial public health concern. Indeed, the majority of a general dental practitioner’s work revolves around the prevention, diagnosis, and management of dental caries and its consequences.

In the military setting, while combat personnel undergo comprehensive pre-enlistment medical screening to ensure fitness for duty, dental evaluation is often limited in scope or entirely absent. As a result, untreated dental diseases, predominantly caries and its sequelae, account for a significant proportion of non-combat medical disqualifications and disruptions to operational readiness [3]. Given this reality, it is unsurprising that military dental services engage not only in the clinical management of caries across the spectrum, encompassing prevention, early diagnosis, and timely intervention, with complications managed either by a young general practitioner in the field or through specialist referral as appropriate, but also in broader efforts to understand treatment needs, workforce planning, and the health economics of caries-related morbidity among military personnel.

In this issue of the *Israel Journal of Health Policy Research*, Levy et al. present their cross-sectional study titled “Intellectual capability and its association with severe dental caries treatment needs in young Israeli adults” [4]. This investigation builds upon the authors’ previous work, which examined the prevalence of dental caries among military recruits [5]. In their prior study, the authors found that socio-demographic factors, including intellectual capability scores (ICS) and socio-economic status (SEC), were significantly associated with dental treatment needs in young Israeli adults. Specifically, lower ICS scores and lower SEC were linked to a greater need for dental treatments, including restorations, endodontic (root canal) therapies, and dental extractions. Using treatment need as a proxy for severe dental caries is justified, as the requirement for endodontic therapy or extraction generally reflects extensive carious progression into the pulp or a tooth deemed non-restorable. Additionally, immigrants were found to have significantly higher treatment needs than non-immigrants. These findings underscore the critical role of socio-economic and cognitive factors in shaping dental health outcomes within this population. The current study further explores the relationship between cognitive ability and the necessity for invasive dental treatments, particularly extractions and endodontic procedures, within a large cohort of conscripts. By analyzing dental records from 21,052 soldiers, the study reveals that lower intellectual capability scores and socio-economic strata are strongly correlated with a higher need for endodontic treatments and extractions. The study also identifies male gender, older age, and non-native Israeli status as additional predictors of greater treatment requirements [4]. These studies offer valuable insights into the determinants of dental morbidity, reflecting broader social, cognitive, and systemic influences on oral health, albeit with some limitations in generalizability due to the male-predominant composition of the study population.

The demonstrated association between lower intellectual capability and increased need for invasive dental treatments aligns with a growing body of literature emphasizing the role of cognitive, educational, and socio-economic factors in health behaviors and access to care [6, 7]. This also underscores the importance of integrating dental care into comprehensive, equitable health promotion strategies, especially for vulnerable or transitioning populations.

A key innovation of these publications lies not only in their methodological rigor and large-scale analysis of Israel Defense Forces (IDF) population data, but also in their dissemination through high-impact international medical and dental journals, extending their relevance well beyond the traditional confines of military medicine. This reflects a growing recognition by researchers, reviewers, and editors alike that the implications of such studies extend beyond the immediate purview of internal military health services. Indeed, due to Israel’s policy of mandatory conscription, the IDF recruit population serves as a near-comprehensive cohort of the nation’s youth, offering a unique opportunity to examine health trends representative of the broader population in late adolescence and early adulthood [8].

As such, these studies should not be viewed merely as internal evaluations for planning military dental services, but rather as contributions to national and even international public health knowledge. The use of military data for broader epidemiologic insight is not unprecedented. For example, the World Health Organization (WHO) has previously cited findings from Israeli military cohorts as representative of national-level data [9, 10]. Another notable example is the study by Levin et al., which investigated the prevalence of aggressive periodontitis among Israeli military recruits [11]. This study was recognized internationally as the only one to apply rigorous epidemiologic methodology to this condition and, as such, has been considered the most reliable estimate of its true global prevalence [12].

Levy et al. rightly conclude that those with lower intellectual capability and lower SES are at higher risk of invasive dental treatments, and thus should be prioritized for preventive interventions [13]. Because the analysis was cross-sectional, one cannot prove intervention on these factors will reduce disease; however, the associations suggest that outreach strategies focusing on cognitively or socially disadvantaged youth could yield improvements. The study’s findings support the need for targeted health education and measures to improve attendance at preventive dental services, as the authors recommend. An important methodological takeaway is that traditional risk assessments for oral health (which often consider SES, diet, oral hygiene) might be enhanced by considering cognitive or educational level as a risk marker. The commentary by the authors hints at this broader approach, and the data provide a strong evidence base for such initiatives.

The multidisciplinary research team of the present paper, led by Solomonov and composed of current and former military dental officers, alongside Prof. Zusman, a distinguished public health scholar, exemplifies the Talmudic principle of safra ve-seyfa (“book and sword”). This expression, representing the integration of intellectual rigor and military service, holds particular relevance in the field of military medicine, where a solid foundation of scientific knowledge and clinical expertise, must be complemented with adaptability, strategic decision-making, and the capacity to perform effectively under challenging and high-pressure conditions.

Thus, the work of Levy et al. illustrates the powerful contribution that military health systems, and military dentists in particular, can make to national and global health understanding. By leveraging large-scale, routinely collected data in a unique population, and by bridging the worlds of clinical dentistry, public health, and operational military medicine, their research embodies the very spirit of *safra ve-seyfa*.

## Data Availability

No datasets were generated or analysed during the current study.

## Abbreviations

ICS: Intellectual capability scores
IDF: Israel Defense Forces
SEC: Socio-economic status
WHO: World Health Organization
